# Gibberellin biosynthesis in *Lotus japonicus* regulates arbuscule distribution, but not overall colonisation by arbuscular mycorrhizal fungi

**DOI:** 10.3389/fpls.2026.1772317

**Published:** 2026-03-20

**Authors:** Edwin Jarratt-Barnham, Giles E. D. Oldroyd

**Affiliations:** 1 Crop Science Centre, University of Cambridge, Cambridge, United Kingdom; 2 Donald Danforth Plant Science Center, St. Louis, MO, United States

**Keywords:** AM symbiosis, arbuscular mycorrhizal fungi (AM fungi), copalyl diphosphate synthase, gibberellins, kaurene oxidase, kaurene synthase, kaurenoic acid oxidase, *Lotus japonicus*

## Abstract

Gibberellins have been reported to play both positive and negative roles in arbuscular mycorrhizal (AM) symbioses. Despite extensive characterisation of the role of DELLAs in AM colonisation, studies of gibberellin function have largely been restricted to chemical interventions. Few studies have examined how disruption to gibberellin biosynthesis affects AM symbioses. To explore this further, we obtained *Lotus japonicus LORE1* transposon insertion mutants in four key gibberellin biosynthetic genes: *CPS, KS, KO*, and *KAO*. Through a characterisation of their developmental phenotypes, we determined that for each gene there is a single homolog which has a major role in gibberellin biosynthesis. We name these genes *CPS1, KS1, KO1*, and *KAO1.* Mutations in these genes affect AM colonisation in the overall distribution of arbuscules, but not in total colonisation levels. These results are consistent with previous studies indicating that DELLAs control the number of cortical cell layers, and therefore regulate the number of cells able to accommodate arbuscules.

## Introduction

Arbuscular mycorrhizal (AM) fungi associate with plants in an ancient symbiosis that emerged over 400 million years ago (Remy et al., 1994; Redecker et al., 2000). The majority of land plants engage in this symbiosis, from which they receive inorganic nutrients and water in exchange for a source of carbon derived from photosynthesis (Luginbuehl and Oldroyd, 2017). Multiple factors influence a plant’s decision to engage with AM fungi, including phosphate availability (Mosse, 1973), and this environmental regulation of the AM symbiosis is thought in part to be controlled by plant hormone levels (Liao et al., 2018).

Gibberellins have emerged as one of the more potent negative regulators of AM symbioses, with the application of gibberellic acid (GA_3_) shown to significantly suppress AM colonisation in many species (El Ghachtouli et al., 1996; Floss et al., 2013; Takeda et al., 2015). Application of GA_3_ appears to have a particularly profound effect on the development and maintenance of arbuscules (Nouri et al., 2021). Gibberellin-deficiency has also been shown to increase AM colonisation: the pea *na-1* mutant, carrying a mutation in *KAURENOIC ACID OXIDASE (KAO)*, showed increased arbuscule abundance (Foo et al., 2013), and petunia lines overexpressing a gibberellin methyltransferase, which converts gibberellins into bioactively inert forms, displayed increased AM colonisation (Nouri et al., 2021). Similarly, application of the gibberellin biosynthetic inhibitor, paclobutrazol, has been reported to increase arbuscule abundance in *Lotus japonicus* (Pimprikar et al., 2016). Thus, gibberellins have been proposed as negative regulators of AM symbioses. This conclusion has been further supported by the finding that *della* mutants, which affect gibberellin signalling, show severe reductions in AM colonisation (Floss et al., 2013; Foo et al., 2013; Yu et al., 2014), whilst wheat lines carrying a degradation-resistant allele of DELLA (*Rht)* display increased levels of AM colonisation (Floss et al., 2013).

This portrait of gibberellins as negative regulators of AM symbioses, however, is not entirely consistent. For instance, application of the gibberellin biosynthesis inhibitor uniconazole significantly reduces arbuscule abundance in *L. japonicus* (Takeda et al., 2015), and the gibberellin-insensitive *gid1* mutant in rice shows no significant changes in AM colonisation (Yu et al., 2014). Furthermore, it has been shown that application of GA_3_ in petunia can elicit the transient expression of key arbuscule marker genes (Nouri et al., 2021). It has also been seen in *L. japonicus* and petunia that AM colonisation increases the expression of genes associated with gibberellin biosynthesis, such as *COPALYL DIPHOSPHATE SYNTHASE (CPS), KAURENE SYNTHASE (KS), KAURENE OXIDASE (KO), KAO*, and gibberellin oxidase genes (Takeda et al., 2015; Nouri et al., 2021). In *L. japonicus*, this results in an overall increase in gibberellin abundance in colonised roots (Takeda et al., 2015). Gibberellin biosynthetic genes also appear to be upregulated in the rice *suppressor of max2 1 (smax1)* mutant (Hull et al., 2021), an important negative regulator of AM symbiosis, with *smax1* mutants displaying increased AM colonisation (Choi et al., 2020). These results demonstrate that the gibberellin signalling pathway interacts substantially with symbiotic signalling, and whilst these increases in gibberellin biosynthetic gene expression and gibberellin abundance may be indicative of negative feedback on AM colonisation, they might, alternatively, suggest a positive role for gibberellins in certain aspects of AM symbiosis.

Therefore, whilst it has been well demonstrated that gibberellins can have a negative impact on AM colonisation, it is also possible that gibberellins play a multifaceted role during AM colonisation, potentially dependent on spatial or temporal factors, as has recently been demonstrated to be important in root nodule symbioses (Drapek et al., 2024). It may also be that some of the transcriptional activity observed for genes associated with gibberellin biosynthesis is associated with the upregulation of alternative biochemical pathways. Gibberellins belong to the labdane-related diterpenoid (LRD) superfamily of metabolites, with over 7,000 known members (Peters, 2010). All LRDs, including the gibberellins, are derived from geranylgeranyl pyrophosphate (GGPP) by the action of class I (KS), class II (CPS), or bifunctional class I/II diterpene synthases (diTPSs) (Gao et al., 2021). The function of CPS and KS homologs can vary, however. For example, in rice, *Os*CPS1 and *Os*CPS2 are responsible for the biosynthesis of *ent-*copalyl diphosphate, the precursor to gibberellins, whilst another homolog, *Os*CPS4, is responsible for the biosynthesis of the enantiomeric *syn*-copalyl diphosphate, which has no role in gibberellin biosynthesis (Otomo et al., 2004). Similarly, in rice, it is *Os*KS1 which contributes to gibberellin biosynthesis, whilst its many homologs have no role in gibberellin biosynthesis, and instead contribute to the biosynthesis of other metabolites that have roles in plant-plant and plant-microbe interactions (Lu et al., 2018). The role of this wider LRD family in AM colonisation is unknown.

To better understand the role of LRD biosynthetic genes in AM colonisation, we adopted a genetic approach targeting multiple steps in the early stages of gibberellin and LRD biosynthesis. We chose the model legume *L. japonicus* for this study, since it has been well-characterised for the role of gibberellin signalling in AM colonisation, has a comparatively small array of gene homologs involved in gibberellin and LRD biosynthesis, and has available genetic materials from the *LORE1* transposon insertion collection (Fukai et al., 2012; Urbański et al., 2012; Małolepszy et al., 2016; Mun et al., 2016). We show that disruption to gibberellin biosynthesis, at multiple steps in the pathway, results in predictable impacts on plant development, and a change in the distribution of arbuscules within root tissues, but had no detected impact on AM root-length colonisation.

## Materials and methods

### *Lotus japonicus* genetic material

*L. japonicus* (Gifu) *LORE1* transposon insertion mutant and wildtype seeds were provided by the National Agriculture and Food Research Organization (*cps1-1*) and Lotus Base (wildtype and all other *LORE1* mutants) (Fukai et al., 2012; Urbański et al., 2012; Małolepszy et al., 2016; Mun et al., 2016) (**Supplementary Table 1**).

### *Lotus japonicus* seed sterilisation, germination, and propagation

All plant growth was conducted in a growth chamber (Conviron Ltd., Canada) with a 16h/8h day/night cycle, a relative humidity of 55%, 130 µW m^-2^ photosynthetically active radiation, and a temperature of 22 °C. *L. japonicus* seeds were scarified with sandpaper then surface sterilised with ethanol solution (70% v/v EtOH and 0.1% w/v sodium dodecyl sulphate (SDS) in sterile ultrapure water), followed by bleach solution (2% v/v NaClO and 0.1% w/v SDS in sterile ultrapure water). Seeds were washed, then left in sterile ultrapure water overnight, being rotated at 60 rpm. Imbibed seeds were placed on 0.8% agar plates and left to germinate for 10 days.

For seed propagation, seedlings were grown in Levington Advance Pot & Bedding M3 Peat Reduced Compost and Levington Advance Seed & Modular F2 Formula Compost (Evergreen Garden Care, UK). Plants were watered with tap water to maintain soil moisture. Gibberellin-deficient lines (*cps1, ks1, ko1, kao1*) were sprayed three times a week with 10 uM gibberellic acid (GA_3_) (Merck Life Science UK Limited, UK), which had been dissolved in ethanol to produce a 1000-fold stock, then diluted in tap water before application. Homozygous transposon insertion mutants were identified by PCR and gel electrophoresis using the *LORE1* primer 5’-CCATGGCGGTTCCGTGAATCTTAGG-3’ and the gene specific primers listed in **Supplementary Table 2**. Mature seed pods were collected from each plant and stored in seed bags in dry conditions at 4 °C until use.

### *Lotus japonicus* arbuscular mycorrhizal colonisation assays

*L. japonicus* arbuscular mycorrhizal (AM) colonisation assays were conducted according to the methods described by Torabi et al., 2021. Namely, closed-topped pots (OS 140 Box/Green Filter, Duchefa, Netherlands) were prepared with 500 g sterile sand (grade 14/25, Aggregate Industries, UK) which was moistened with 40 mL modified B&D medium containing 200 µM P_i_ (**Supplementary Table 3**). Subsequently, 3000 spores of *Rhizophagus irregularis* (Premier Tech, Canada) were applied in 10 mL sterile water, and spread evenly throughout the growth medium. Six seedlings, germinated as described above, were placed in each pot and the pots were then sealed with their lid.

### Staining and quantification of arbuscular mycorrhizal root-length colonisation

At the point of harvest, roots of a single plant were chopped into approximately 1.5 cm long sections, mixed thoroughly, and a sample placed into 10% KOH. Samples were subsequently stained as described by Evangelisti et al., 2021, using Clearsee (Kurihara et al., 2015; Evangelisti et al., 2021). Namely, sampled roots were heated at 96 °C for 10 minutes in the 10% KOH, washed in 5% acetic acid, and stained by heating roots in Sheaffer Skrip staining solution (5% v/v Sheaffer Skrip black ink (Sheaffer, USA) and 5% v/v acetic acid in sterile ultrapure water) to 96 °C for 10 minutes. Roots were washed with RO water and cleared with Clearsee solution (25% w/v Urea, 10% w/v xylitol, and 15% w/v sodium deoxycholate in sterile ultrapure water) for 30 seconds. Roots were then mounted onto a microscope slide (ThermoFisher, USA) in mounting medium (20% v/v glycerol, 50 mM Tris-HCl, pH 7.5, and 0.1% v/v Tween-20 in sterile ultrapure water). At all times, it was ensured that root samples and root pieces were selected randomly by thorough mixing of root samples and unbiased selection. The slide was covered with a coverslide (Marienfeld, Germany), and sealed using top-coat nail polish.

All fungal structure quantification was carried out according to the methods described by Torabi et al., 2021 and Jarratt-Barnham et al., 2024, under a total 200 x magnification. 100 fields-of-view were sampled per biological replicate with 10 fields of view per root piece. Each field-of-view was scored for the presence or absence of fungal structures using AMScorer (Jarratt-Barnham et al., 2024). Data analysis was performed by AMReader (Jarratt-Barnham et al., 2024).

### Assessment of arbuscule distribution within roots

To quantify the radial distribution of arbuscules, a systematic transect-based approach was employed. Transects, each 50 μm wide, were placed perpendicular to the longitudinal root axis, originating at the vasculature and extending to the epidermis. Transects were placed systematically in adjacent fields-of-view in well-colonised root regions. For each transect, three distances were measured: the distance from the vasculature to the first arbuscule boundary, the distance from the vasculature to the final arbuscule boundary, and the distance from the vasculature to the epidermis. To account for variance in root width, all measurements were normalised to the distance between the vasculature and epidermis.

### Phylogenetic analysis

To produce each phylogeny, the protein sequence of a representative gene was submitted to tblastn against the annotated coding sequences of fourteen genomes (**Supplementary Table 4**) (Ouyang et al., 2007; Sato et al., 2008; Tang et al., 2014; Hirsch et al., 2016; Mun et al., 2016; Beier et al., 2017; Bowman et al., 2017; Cheng et al., 2017; Mascher et al., 2017; Lang et al., 2018; McCormick et al., 2018; Hosmani et al., 2019; Kreplak et al., 2019; Hufnagel et al., 2020; Kamal et al., 2020; Radhakrishnan et al., 2020) (ncbi-blast-2.10.1+ package available from https://ftp.ncbi.nlm.nih.gov/blast/executables/blast+/LATEST/, last accessed 08/05/2021) (Camacho et al., 2009). Hits were filtered such that e > 10^–4^ and percentage cover > 30%, then the coding sequences of the top 200 remaining hits were submitted to sequence alignment by MAFFT using default parameters (available from https://mafft.cbrc.jp/alignment/software/, last accessed 08/05/2021) (Katoh and Standley, 2013). IQTREE version 1.x was used to construct phylogenies using default parameters with a bootstrap value of 1000 (available from http://www.iqtree.org/, last accessed 08/05/2021) (Minh et al., 2020).

### Statistical analysis

Unless otherwise stated, all sample sizes described in this study refer to biological replicates, where each biological replicate is taken from a single plant. Statistical analysis was conducted in R (v 4.2.2). Where suitable, a one-way ANOVA was conducted to assess whether there were statistically significant differences observed between groups. If differences were observed this was followed by the *post-hoc* Tukey HSD test. Otherwise, a Kruskal-Wallis test was applied, followed by *post-hoc* Wilcoxon Rank Sum tests and *post-hoc* Dunn tests. In all cases, statistical significance was assessed according to the threshold p < 0.05. Specific information is described in the figure legend for all experiments.

## Results

### Characterisation of the gibberellin biosynthetic pathway in *Lotus japonicus*

**Figure 1:**
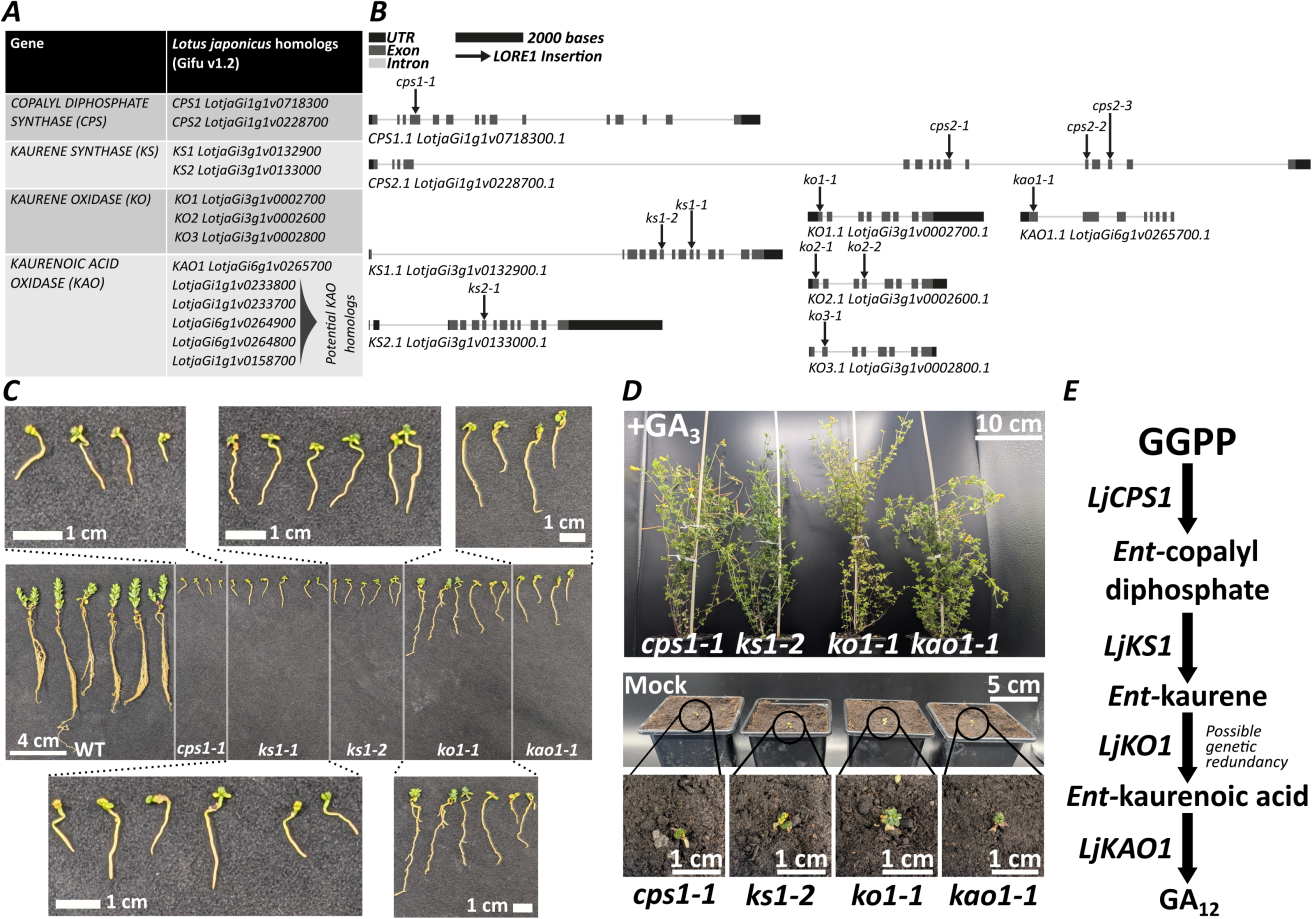
*L. japonicus* mutant growth phenotypes elucidate those homologs necessary for gibberellin biosynthesis. **(A)** Homologs of *CPS, KS, KO*, and *KAO* identified in the *L. japonicus* genome (Gifu v1.2). **(B)** Gene structures of the identified homologs, and the locations of *LORE1* transposon insertions used in this study. **(C)** Representative images of the growth phenotypes of *cps1, ks1, ko1*, and *kao1* mutants, displaying severe growth phenotypes. Plants were grown under conditions described for arbuscular mycorrhizal colonisation assays and imaged at 9 weeks old. **(D)** Representative images showing the rescue of mutant plant growth phenotypes by gibberellin A_3_ application, compared with plants treated with the mock control. Plants were grown under conditions described for seed propagation and imaged at 26 weeks old. Plants were supplemented with 10 μM GA_3_, or a mock treatment, three times a week. **(E)** The proposed gibberellin biosynthetic pathway in *L. japonicus*.

The earliest stages of gibberellin biosynthesis involve four key enzymes: CPS, KS, KO, and KAO. Through phylogenetic analyses, we were able to determine that *L. japonicus* has a comparatively small complement of these biosynthetic genes (**Figure 1A**, **Supplementary Figures 1**-**4**). Peculiarly, we identified six *KAO* homologs in the Gifu v1.2 genome, compared to only a single *KAO* homolog in the MG20 genome. We reasoned that either there had been successive duplications since these ecotypes diverged, or that these additional homologs may be an artefact of genome assembly. Favouring the latter, we considered these additional *KAO* homologs putative only.

We subsequently examined the expression patterns of all the identified homologs through data from the Lotus Base Gene Atlas (Mun et al., 2016). In brief, these data show that *CPS1, CPS2, KS1, KS2, KO1, KO3*, and *KAO1* are all expressed to a significant degree in at least one of the tissues examined, with *KO2* weakly expressed in all tissues (**Supplementary Figure 5**). By comparison, the *KAO* homologs which we believe are artefacts of genome assembly (putative *KAO* homologs) are poorly expressed, with expression only very weakly detectable in the pod. Both *CPS1* and *CPS2* are predominantly expressed in root tissues, though expression can also be observed in other tissues. *KS1* expression is found throughout all the tissues examined, whilst *KS2* expression appears to be restricted to root tissues. *KO1* appears to be the most strongly expressed *KO* homolog in all tissues, whilst *KO3* expression is predominantly found in the seed. *KAO1* expression is greatest in the seed, though expression is observed throughout all the tissues examined.

We subsequently collected knock-out mutants, where available, in all target genes from the *LORE1* transposon insertion library (Fukai et al., 2012; Urbański et al., 2012; Małolepszy et al., 2016; Mun et al., 2016) (**Figure 1B**). No *LORE1* insertion mutants were available for the putative *KAO1* homologs, which we interpret as further evidence that these sequences are artefacts of the genome assembly process.

From the *LORE1* seed provided, we obtained homozygous mutant lines and then proceeded to characterise their developmental phenotypes. We determined that *cps1, ks1, ko1*, and *kao1* mutants display severe dwarf phenotypes with greatly impaired shoot and root development, including reduced shoot length, reduced root length, increased root width, and reduced biomass (**Figures 1C, D**; **Supplementary Figures 6A–D****).** The developmental impact was slightly less severe in the *ko1-1* mutant compared with the other mutants (**Figure 1C**; **Supplementary Figures 6A–D**). This may suggest that either one, or both, of *KO2* and *KO3* may partially contribute to gibberellin biosynthesis. Alternatively, it may be that the *LORE1* insertion has not fully eliminated the production or function of the KO1 protein. Lack of clear genetic redundancy in *kao1-1* mutants supported our prior hypothesis that there is only one copy of *KAO* in the *L. japonicus* genome which plays a major role in gibberellin biosynthesis. We did not observe any significant growth phenotypes amongst the mutants of other gene homologs, and we did not find any consistent AM colonisation phenotypes sufficient to demand further investigation (**Supplementary Figures 6A–D**, **7**). Despite significant expression of both *CPS2* and *KS2* in root tissue, therefore, these genes do not appear to alleviate the severity of growth phenotypes. This suggests they may have functions other than gibberellin biosynthesis.

To confirm that the dwarfism in our mutant lines was due to gibberellin-deficiency, we sought to rescue their phenotypes through gibberellin application. Continued application of GA_3_ was sufficient for all dwarf mutants to complete their lifecycle and produce viable seed, in contrast to plants which received mock treatments, which remained dwarf, even across a period of many months (**Figure 1D**). Together, these results demonstrate developmental functions consistent with gibberellin-deficiency in *L. japonicus*, would indicate that we have successfully characterised multiple independent gibberellin-deficient mutants, and allow us to elucidate those gene homologs which play major roles in gibberellin biosynthesis in *L. japonicus* (**Figure 1E**).

### Gibberellin-deficiency has no measurable effect on AM root-length colonisation in *Lotus japonicus*

**Figure 2:**
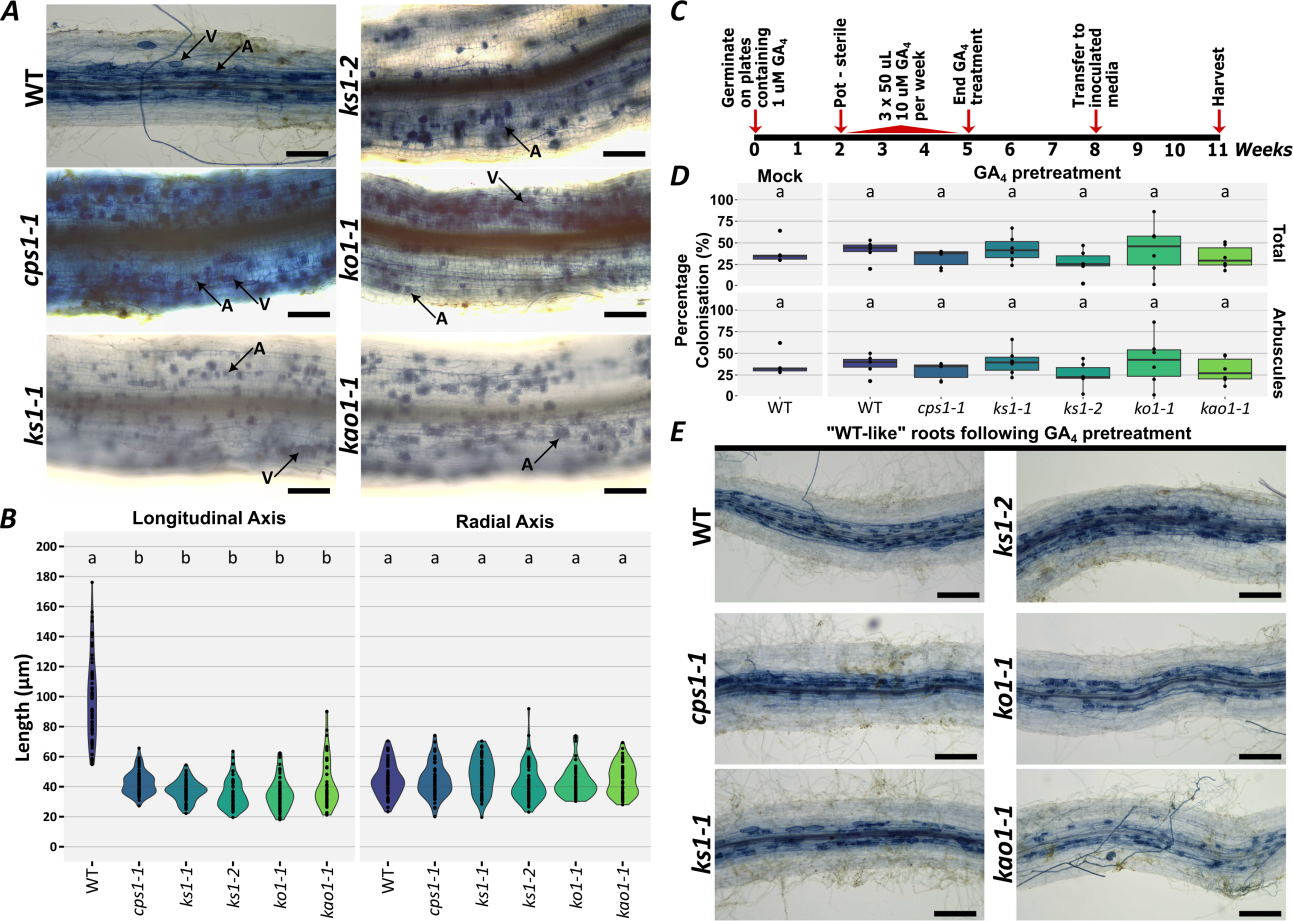
Gibberellin biosynthetic genes play little role in establishing AM root-length colonisation in *L. japonicus*. **(A)** Representative images of AM root-length colonisation within roots of wildtype (WT) and gibberellin-deficient mutants. Scale bar represents 200 μm. **(B)** WT root cells were more elongated in the longitudinal axis compared to gibberellin-deficient mutants, but not significantly different in the radial axis. (Tukey HSD < 0.05) (n = 60 measurements from 3 biological replicates, 20 cells measured from each replicate. Each replicate represents a single plant). Statistics were calculated independently for each panel. **(C)** An experimental time-course for the data displayed in **(D, E)**. **(D)** Root-length colonisation of gibberellin-deficient mutants which received pre-treatment with gibberellin A_4_. No statistical differences were observed (Kruskal Wallis Test, p = 0.57 for percentage total root-length colonisation and p = 0.63 for percentage arbuscule root-length colonisation) (n ≥ 5 biological replicates. Each replicate represents a single plant). Statistics were calculated independently for percentage total and percentage arbuscule root-length colonisation. **(E)** Representative images of arbuscular mycorrhizal root-length colonisation within roots of the wildtype and gibberellin-deficient mutants, where root development had been rescued by application of GA_4_.

Having identified those genes which are key for gibberellin biosynthesis, we tested whether gibberellin-deficiency has any impact on AM colonisation. First, we grew the mutant seed in the absence of any gibberellin supplement. We thereby found that *cps1, ks1, ko1*, and *kao1* mutants all displayed intraradical colonisation and we observed typical fungal structures, such as arbuscules and vesicles, throughout the root system (**Figure 2A**).

Again, we observed that the roots of our mutants were significantly thicker than wild type (**Figure 2A**; **Supplementary Figure 6C**). This could not be readily explained by changes in cell size. In the *cps1, ks1, ko1*, and *kao1* mutants, cell lengths were reduced in the longitudinal axis, but unchanged in the periclinal axis (**Figure 2B**). Considering results from Fonouni-Farde et al., 2019, who found an increase in cortical cell layers in gibberellin-deficient *Medicago truncatula*, we infer that our gibberellin-deficient *L. japonicus* lines likely have an increased number of cortical cell layers.

**Figure 3:**
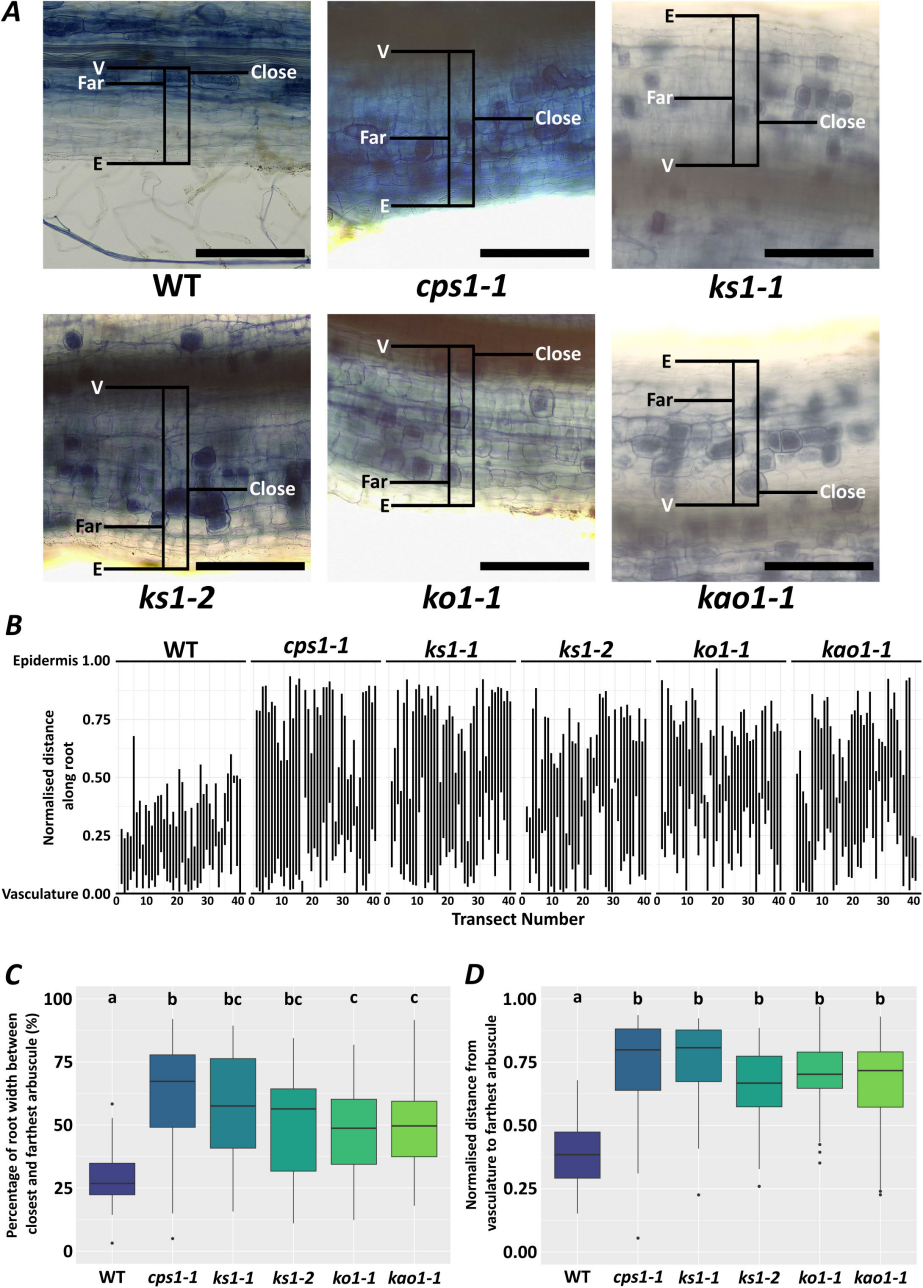
Arbuscule distribution is broadened in gibberellin-deficient *L. japonicus* lines. **(A)** Representative images of AM root-length colonisation within roots of wildtype (WT) and gibberellin-deficient mutants. Scale bar represents 200 μm. Images are zoomed-in panels of **Figure 2A**. Overlay illustrates the positioning of a transect from root vasculature (V) to epidermis (E). For each transect, the position of the closest (close) and farthest (far) arbuscule was recorded. Distances were normalised to the distance between the vasculature and epidermis. **(B)** An illustration of the distribution of arbuscules found in WT and mutant lines. Each vertical line represents the distance between the closest (close) and furthest (far) arbuscule in each transect. Distances were normalised to the distance between the vasculature and epidermis. **(C)** The distance between the closest (close) and farthest (far) arbuscule, as a percentage of the root width measured. Significance groups were calculated and shown (Dunn Test with Holm adjustment, p < 0.05) (n = 40 transects collected from 3 biological replicates). **(D)** The distance from the vasculature to the farthest arbuscule, normalised to the root width measured. Significance groups were calculated and shown (Dunn Test with Holm adjustment, p < 0.05) (n = 40 transects collected from 3 biological replicates).

Strikingly, whereas arbuscules form within wildtype plants in discrete cell layers, previously demonstrated to be the inner cortex (Demchenko et al., 2004), *cps1, ks1, ko1*, and *kao1* mutants showed arbuscules across a greater width of the root (**Figure 2A**; **Figure 3**). This would be consistent with an expansion in cortical cell layers, and would indicate that these additional cell layers remain competent for arbuscule formation. It was recently demonstrated that DELLA controls the number of inner cortical cells which are competent for arbuscule formation at the stem cell niche of *M. truncatula* (An et al., 2025). Our results would suggest that gibberellin-deficiency also increases the number of cell layers competent for arbuscule formation, presumably by a similar mechanism through DELLA stabilisation.

We subsequently sought to quantify root-length colonisation in these lines; however, standard protocols were not suitable due to the severe nature of the mutant root developmental phenotype. To overcome this, we set up new experiments in which we rescued plant growth by applying GA_4_, restoring “WT-like” shoot and root growth. We then allowed time for the effect of these gibberellins to dissipate, as could be monitored by the re-emergence of dwarfing phenotypes in the shoot, and then transplanted the plants into fresh growth medium which had been inoculated with AM fungi (**Figure 2C**). This allowed us to produce plants which were deficient in gibberellins, as evidenced by re-emergence of plant dwarf phenotypes, but which had sufficient root material for a standard AM colonisation assay. Through this approach, we sought to limit the possible pleiotropic effects that may arise from the severe developmental phenotype of gibberellin-deficient mutants, and remove any impact of the gibberellin-pretreatment, though it is not possible to eliminate either variable entirely. Quantification of AM colonisation found no evidence to suggest that gibberellin-deficiency led to significant differences in root-length colonisation (**Figure 2D**).

In these experiments, we also saw no differences between WT plants which had been pretreated with gibberellin A_4_ and those which had received a mock treatment (**Figure 2D**). Given the very strong negative impact gibberellin application has on AM colonisation (El Ghachtouli et al., 1996; Floss et al., 2013; Takeda et al., 2015; Nouri et al., 2021), we infer that the effects of our earlier gibberellin supplementation had not significantly interfered with our colonisation assay. We could also observe that the distribution of arbuscules within the “WT-like” roots of gibberellin-deficient mutants was similar to their distribution in WT plants, where arbuscules are found close to the vasculature (**Figure 2E**; **Supplementary Figure 8**). This would indicate that the changes in the distribution of arbuscules observed previously (**Figure 2A**; **Figure 3**) are predetermined by root development, and are not regulated by a gibberellin-mediated signal during the course of AM colonisation. Overall, there was no evidence to suggest that gibberellin-deficiency has significant impacts on AM root-length colonisation in *L. japonicus*, beyond the impacts associated with changes in plant physiology.

## Discussion

AM symbiosis requires a complex and tightly regulated series of interactions between plants and AM fungi. Plant hormones play a major role throughout this interaction, with gibberellins probably the most extensively studied. Genetic analyses have shown that DELLA proteins are essential for normal mycorrhizal colonisation, especially for appropriate arbuscule development (Foo et al., 2013; Floss et al., 2016; Jin et al., 2016; Pimprikar et al., 2016). Since DELLAs are negatively regulated by gibberellins (Hernández-García et al., 2021), it has been inferred that the requirement for DELLAs reflects a function of gibberellins. However, DELLAs play a role in many signalling processes (Hernández-García et al., 2021), and can be regulated independently of gibberellins (Rana et al., 2025). Likewise, gibberellins can have functions independent of DELLAs (Ito et al., 2018). To understand the role of gibberellins in AM symbiosis, therefore, it is important to assess the role of gibberellins more directly, especially in light of the absence of an AM colonisation phenotype in the gibberellin receptor mutant *gid1* of rice (Yu et al., 2014).

Direct analysis of gibberellin function, however, has mostly been limited to chemical interventions, whether through the application of gibberellins, or the application of gibberellin biosynthetic inhibitors (El Ghachtouli et al., 1996; Floss et al., 2013; Foo et al., 2013; Takeda et al., 2015; Pimprikar et al., 2016). Throughout, it has been shown that exogenous application of gibberellins suppresses AM colonisation (El Ghachtouli et al., 1996; Floss et al., 2013; Foo et al., 2013; Takeda et al., 2015), but genetic approaches assessing the effect of gibberellin-deficiency have been very limited. The exception to this is the pea *na-1 (kao)* mutant, which showed greater arbuscule abundance, but no significant effect on total root-length colonisation (Foo et al., 2013). In this mutant, gibberellin application suppressed AM colonisation independently of any anatomical changes caused by gibberellin-deficiency, suggesting a direct effect on symbiotic signalling (Foo et al., 2013).

Here, we have obtained multiple independent mutants at multiple steps in gibberellin biosynthesis in *L. japonicus*. These mutants all display phenotypes associated with gibberellin-deficiency in other species. This includes reduced shoot length, reduced root length, increased root width, and reduced cell elongation along the longitudinal axis (Thomas et al., 2005). The dwarfism of *cps1, ks1, ko1* and *kao1* mutants was rescued by gibberellin application. Whilst we have not quantified gibberellin abundance directly, we infer that we have obtained *bona fide* gibberellin biosynthesis mutants, and that the gibberellin biosynthesis pathway of *L. japonicus* mirrors that found in other well-characterised species, such as *Arabidopsis* and rice (Hedden, 2020). This allowed us to assess the effect of gibberellin-deficiency on AM root-length colonisation in *L. japonicus*, and compare this to other studies in the same species.

The growth phenotypes in our *cps1, ks1, ko1*, and *kao1* mutants were very severe, however this did not seem to impair either intraradical colonisation or formation of arbuscules and vesicles. This would suggest that physiological gibberellin levels are not required for AM colonisation. When we rescued plant growth by pre-treatment with GA_4_, we also found no evidence to suggest that gibberellin-deficiency had an impact on AM root-length colonisation. In pea, rice, and now *L. japonicus*, therefore, at least in the conditions studied, severe disruptions to either gibberellin biosynthesis or gibberellin signalling have all been found to have no impact on total AM root-length colonisation (Foo et al., 2013; Yu et al., 2014). These results would suggest that physiological levels of gibberellins are not restricting AM root-length colonisation, however we can never fully separate such results from substantial developmental phenotypes, and there may still be pleiotropic effects.

Our findings do not support the conclusion of Takeda et al., 2015, that the strong reduction in AM colonisation in *L. japonicus* achieved by uniconazole is due to gibberellin-deficiency. Given this, we believe it is important to consider whether the reduced colonisation seen by Takeda et al. might have been caused by off-target effects of uniconazole, potentially including impacts on cytokinin biosynthesis and abscisic acid catabolism (Saito et al., 2006; Sasaki et al., 2013). We consider this plausible, particularly since another gibberellin biosynthetic inhibitor, paclobutrazol, was not found to reduce AM root-length colonisation (Pimprikar et al., 2016). It remains possible, however, that partial reductions in gibberellin abundance might facilitate an increase in AM root-length colonisation, which could explain the result seen in petunia (Nouri et al., 2021).

In our dwarf mutants we observed an expansion in the distribution of arbuscules across the root. This result is consistent with a recent study in *M. truncatula*, which demonstrated that the number of inner cortical cells competent for arbuscule formation is controlled by DELLA at the root stem cell niche (An et al., 2025). Our results would suggest that gibberellin biosynthesis also plays a key role in regulating the identity of those cells competent for arbuscule formation, likely through the stabilisation of DELLAs.

Overall, while it has been demonstrated that applying excess gibberellins greatly impacts arbuscule frequency, likely explained by their impact on DELLAs (El Ghachtouli et al., 1996; Floss et al., 2013; Takeda et al., 2015; Nouri et al., 2021), our results would suggest that physiological levels of gibberellins do not greatly restrict, and are not required for root-length AM colonisation in *L. japonicus*.

## Data Availability

The original contributions presented in the study are included in the article/**Supplementary Material**. Further inquiries can be directed to the corresponding author.

## Supplementary material

Supplementary Figure 1 Phylogeny of COPALYL DIPHOSPHATE SYNTHASE (CPS). Dicot species are coloured in teal, and monocot species are coloured in orange. The gene ID used as a query sequence for the initial tblastn query is identified with a blue circle. Bootstrap values are displayed for each branch. Insert shows the full unrooted phylogeny from which the CPS clade was identified.

Supplementary Figure 2 Phylogeny of *KAURENE SYNTHASE (KS).* Dicot species are coloured in teal, and monocot species are coloured in orange. The gene ID used as a query sequence for the initial tblastn query is identified with a blue circle. Bootstrap values are displayed for each branch. Insert shows the full unrooted phylogeny from which the *KS* clade was identified.

Supplementary Figure 3 Phylogeny of *KAURENE OXIDASE (KO).* Dicot species are coloured in teal, monocot species are coloured in orange, and other species are coloured in blue. The gene ID used as a query sequence for the initial tblastn query is identified with a blue circle. Bootstrap values are displayed for each branch. Insert shows the full unrooted phylogeny from which the *KO* clade was identified.

Supplementary Figure 4 Phylogeny of KAURENOIC ACID OXIDASE (KAO). Dicot species are coloured in teal, monocot species are coloured in orange, and other species are coloured in blue. The gene ID used as a query sequence for the initial tblastn query is identified with a blue circle. Bootstrap values are displayed for each branch. Insert shows the full unrooted phylogeny from which the KAO clade was identified.

Supplementary Figure 5 Normalised expression of *COPALYL DIPHOSPHATE SYNTHASE (CPS), KAURENE SYNTHASE (KS), KAURENE OXIDASE (KO)*, and *KAURENOIC ACID OXIDASE (KAO)* homologs as recorded in the Lotus Base Gene Atlas (Mun et al., 2016). Data is shown from the following tissues: leaf, root, mature flower, pod, and seed. Letters display statically significant differences between groups (Tukey HSD, p < 0.05, n = 3, see Lotus Base for further information). Statistics were calculated independently for each gene.

Supplementary Figure 6 Characterisation of shoot and root growth phenotypes of *L. japonicus* mutants examined in this study. **(A)** Shoot length of dwarf (left panel and central panel) and non-dwarf (right panel) mutant lines. Significance groups were calculated for each panel (Tukey HSD, p < 0.05) (n ≥ 5 biological replicates. Each replicate represents a single plant). **(B)** Root length of dwarf (left panel and central panel) and non-dwarf (right panel) mutant lines. Significance groups were calculated for each panel (Tukey HSD, p < 0.05) (n ≥ 5 biological replicates. Each replicate represents a single plant). **(C)** Root width of dwarf (left panel and central panel) and non-dwarf (right panel) mutant lines. For each plant, root width was determined as the average of 10 individual regions, randomly selected along the root length. Significance groups were calculated for each panel (Tukey HSD, p < 0.05) (n ≥ 5 biological replicates. Each replicate represents a single plant). **(D)** Fresh root and shoot biomass of dwarf (left panel and central panel) and non-dwarf (right panel) mutant lines. Significance groups were calculated for each panel (Tukey HSD, p < 0.05) (n ≥ 5 biological replicates. Each replicate represents a single plant).

Supplementary Figure 7 Phenotypes of *cps2*, *ks2, ko2* and *ko3* mutants **(A)** Representative images of the growth phenotypes of *cps1*, *ks1*, *ko1*, and *kao1* mutants, displaying severe growth phenotypes, compared with mutants in their respective gene homologs. Plants were grown under conditions for arbuscular mycorrhizal colonisation assays and imaged at 6 weeks old. **(B)** Root-length colonisation of *cps2* and *ks2* mutants. Plants were assessed at 4 and 5 weeks post-inoculation. Significance groups were calculated for each panel (Wilcoxon Rank Sum Test, p < 0.05) (n ≥ 4 biological replicates. Each replicate represents a single plant). Statistics were calculated independently for percentage total and percentage arbuscule root-length colonisation, and independently for each timepoint. **(C)** Root-length colonisation of *ko2* and *ko3* mutants. Plants were assessed at 4 weeks post-inoculation. No statistical differences were observed (Kruskal Wallis Test, p = 0.26 for percentage total root-length colonisation and p = 0.32 for percentage arbuscule root-length colonisation) (n ≥ 5 biological replicates. Each replicate represents a single plant). Statistics were calculated independently for percentage total and percentage arbuscule root-length colonisation.

Supplementary Figure 8 Arbuscule distribution is restored in gibberellin-deficient roots pretreated with gibberellin A_4_. **(A)** An illustration of the distribution of arbuscules found in WT and mutant lines following pretreatment with gibberellin A_4_. Each vertical line represents the distance between the closest (close) and furthest (far) arbuscule in each transect. Distances were normalised to the distance between the vasculature and epidermis. **(B)** The distance between the closest (close) and farthest (far) arbuscule, as a percentage of the root width measured. No statistically significant differences were observed (Kruskal-Wallis test, p = 0.65) (n = 40 transects collected from ≥3 biological replicates). **(C)** The distance from the vasculature to the farthest arbuscule, normalised to the root width measured. No statistically significant differences were observed (Kruskal-Wallis test, p = 0.11) (n = 40 transects collected from ≥3 biological replicates).
