# Supplementary figures and images for: Gibberellin biosynthesis in Lotus japonicus regulates arbuscule distribution, but not overall colonisation by arbuscular mycorrhizal fungi
Source: Front Plant Sci. 2026 Mar 20;17:1772317. doi: 10.3389/fpls.2026.1772317 (PMC13047161; doi:10.3389/fpls.2026.1772317)

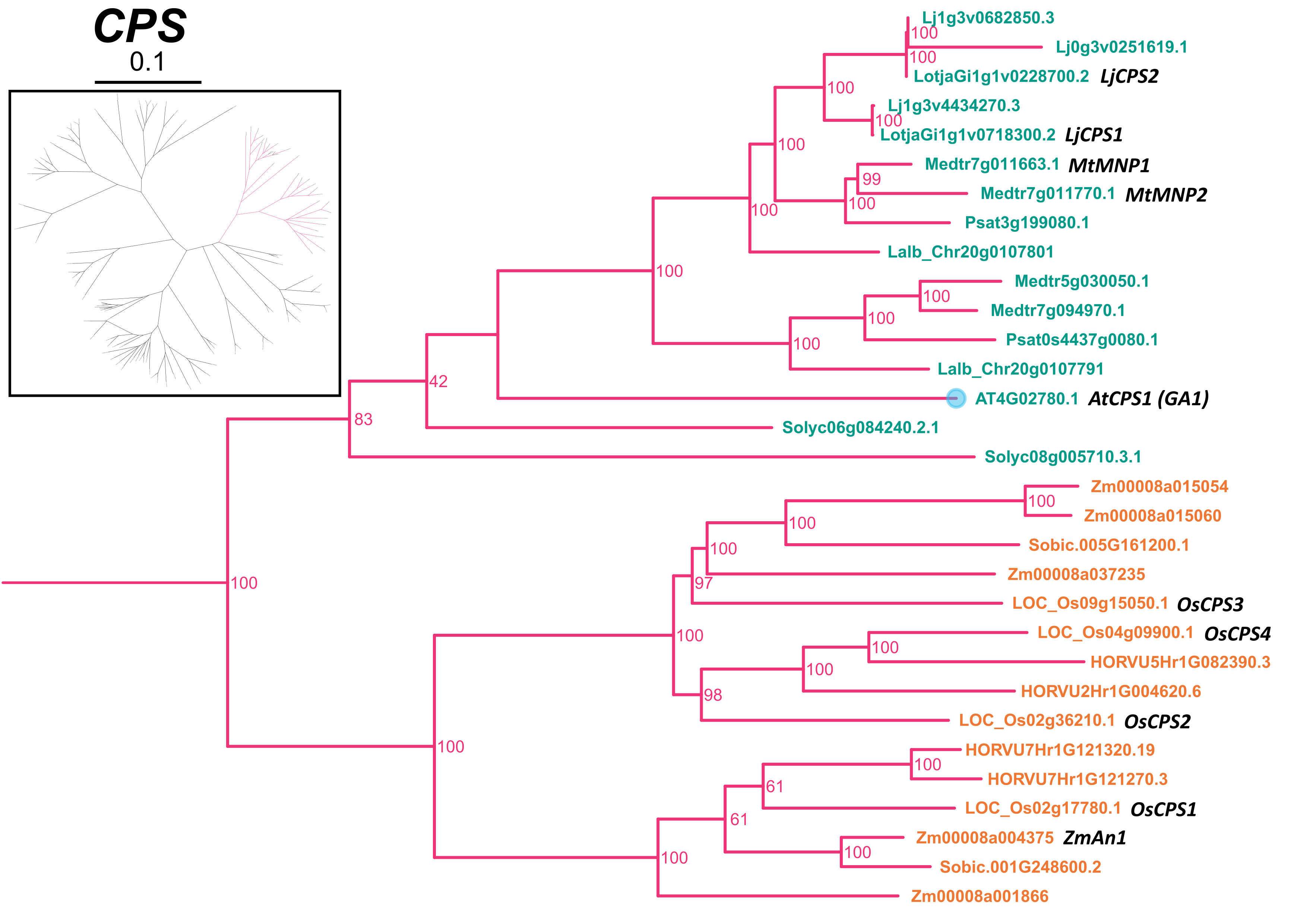

Supplement: Supplementary Figure 1 — Phylogeny of COPALYL DIPHOSPHATE SYNTHASE (CPS). Dicot species are coloured in teal, and monocot species are coloured in orange. The gene ID used as a query sequence for the initial tblastn query is identified with a blue circle. Bootstrap values are displayed for each branch. Insert shows the full unrooted phylogeny from which the CPS clade was identified. [file Image1.tiff]

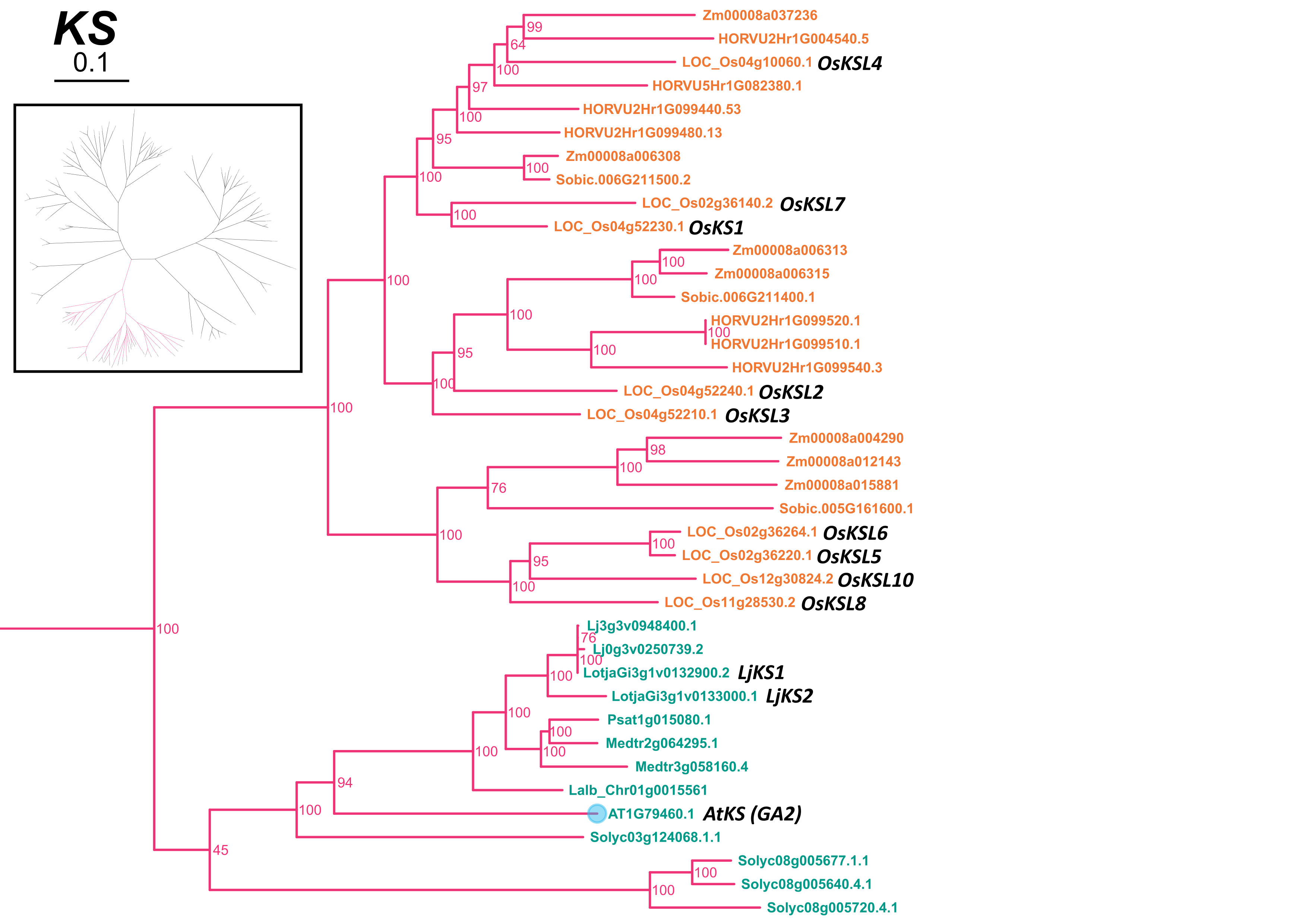

Supplement: Supplementary Figure 2 — Phylogeny of KAURENE SYNTHASE (KS). Dicot species are coloured in teal, and monocot species are coloured in orange. The gene ID used as a query sequence for the initial tblastn query is identified with a blue circle. Bootstrap values are displayed for each branch. Insert shows the full unrooted phylogeny from which the KS clade was identified. [file Image2.tiff]

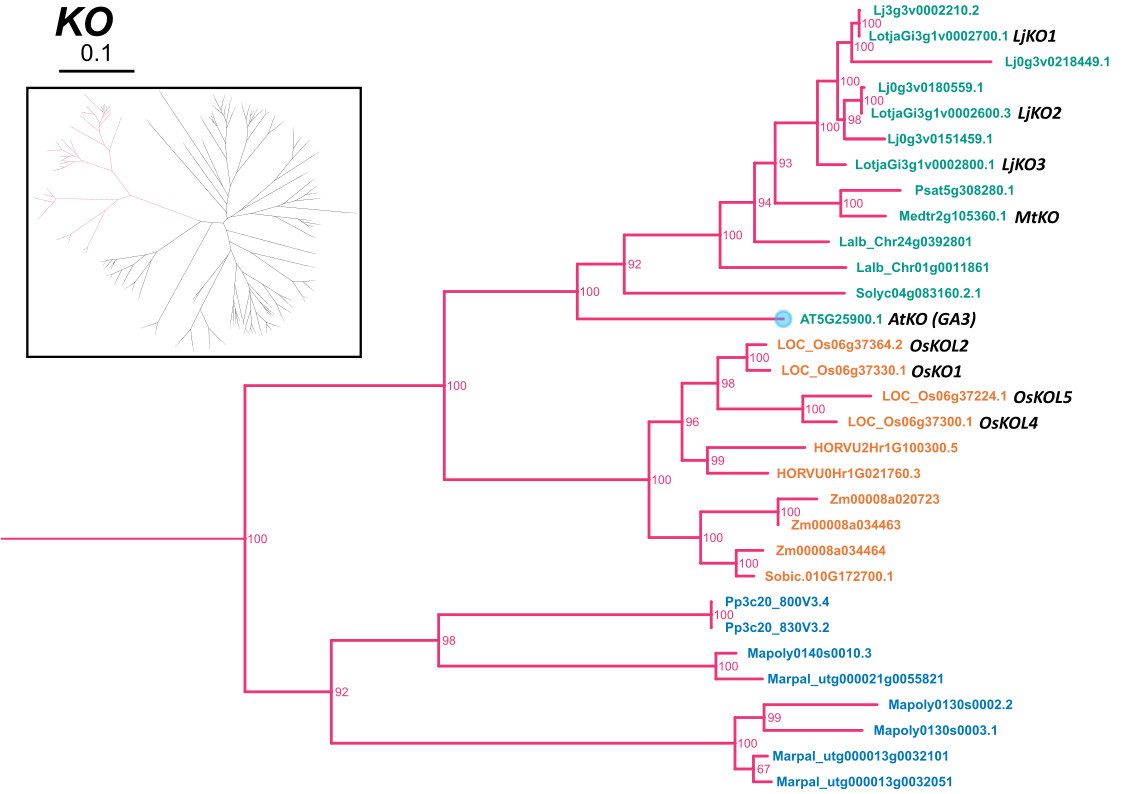

Supplement: Supplementary Figure 3 — Phylogeny of KAURENE OXIDASE (KO). Dicot species are coloured in teal, monocot species are coloured in orange, and other species are coloured in blue. The gene ID used as a query sequence for the initial tblastn query is identified with a blue circle. Bootstrap values are displayed for each branch. Insert shows the full unrooted phylogeny from which the KO clade was identified. [file Image3.tiff]

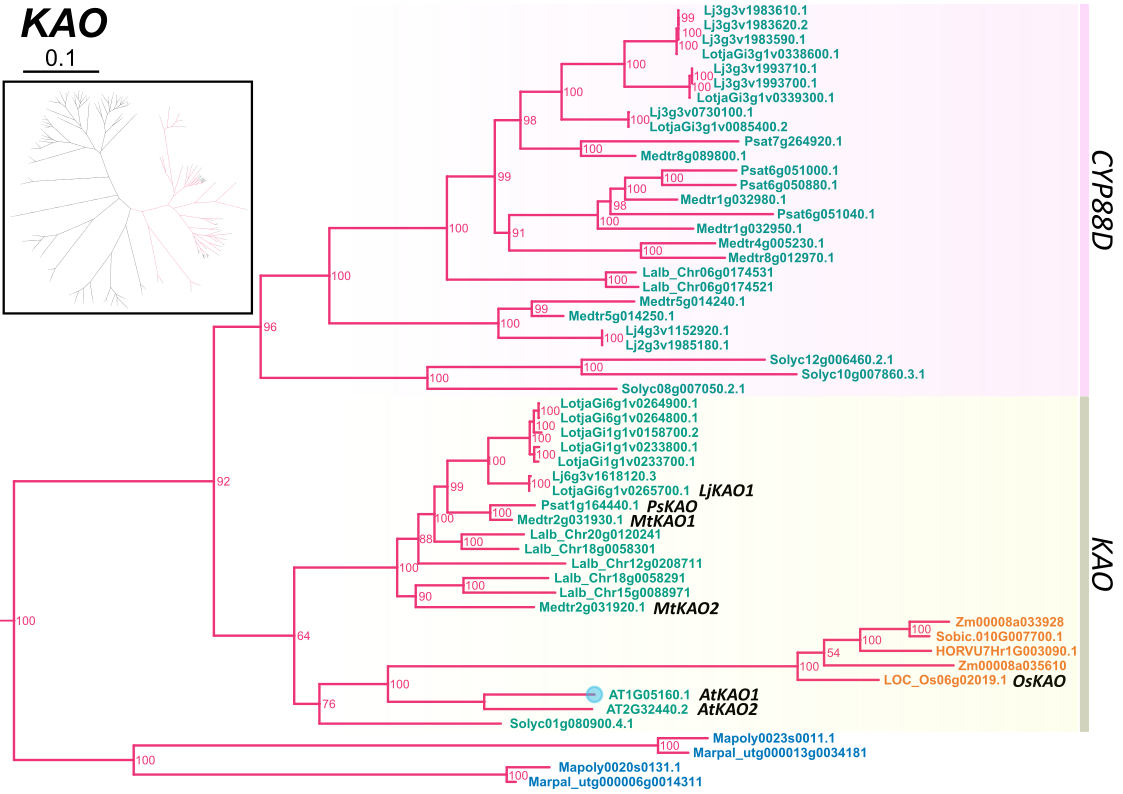

Supplement: Supplementary Figure 4 — Phylogeny of KAURENOIC ACID OXIDASE (KAO). Dicot species are coloured in teal, monocot species are coloured in orange, and other species are coloured in blue. The gene ID used as a query sequence for the initial tblastn query is identified with a blue circle. Bootstrap values are displayed for each branch. Insert shows the full unrooted phylogeny from which the KAO clade was identified. [file Image4.tiff]

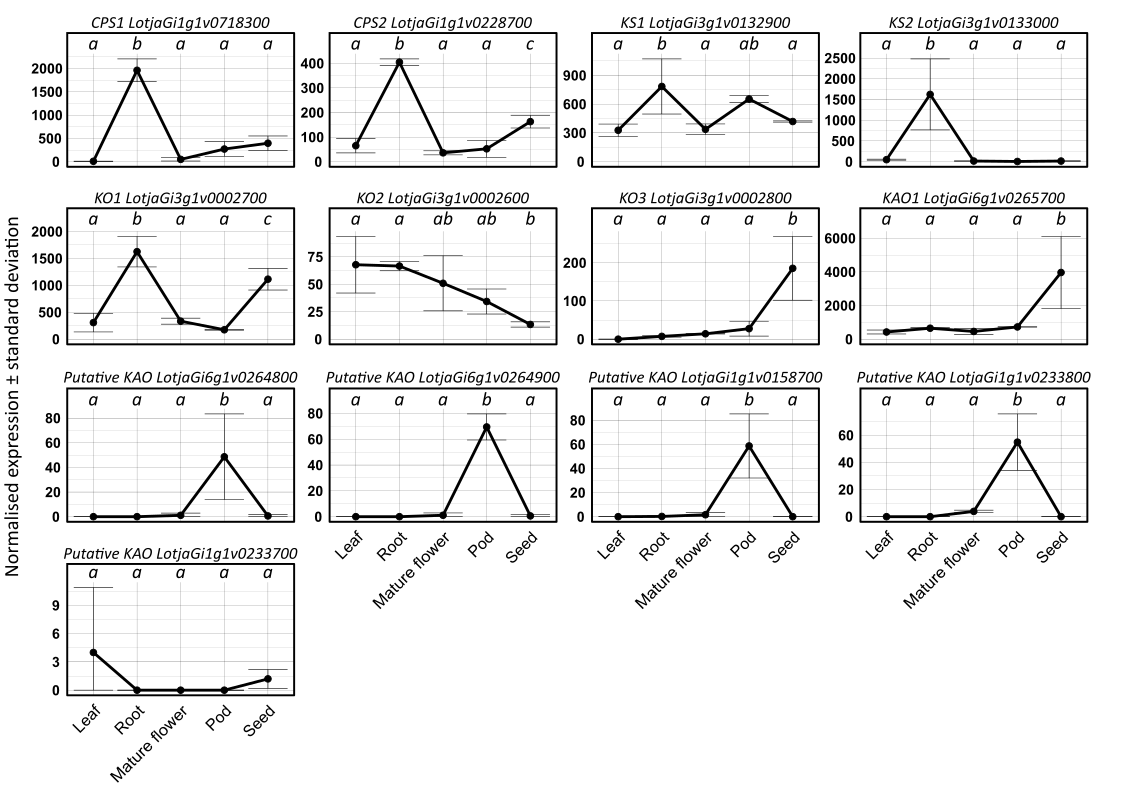

Supplement: Supplementary Figure 5 — Normalised expression of COPALYL DIPHOSPHATE SYNTHASE (CPS), KAURENE SYNTHASE (KS), KAURENE OXIDASE (KO), and KAURENOIC ACID OXIDASE (KAO) homologs as recorded in the Lotus Base Gene Atlas (Mun et al., 2016). Data is shown from the following tissues: leaf, root, mature flower, pod, and seed. Letters display statically significant differences between groups (Tukey HSD, p < 0.05, n = 3, see Lotus Base for further information). Statistics were calculated independently for each gene. [file Image5.tiff]

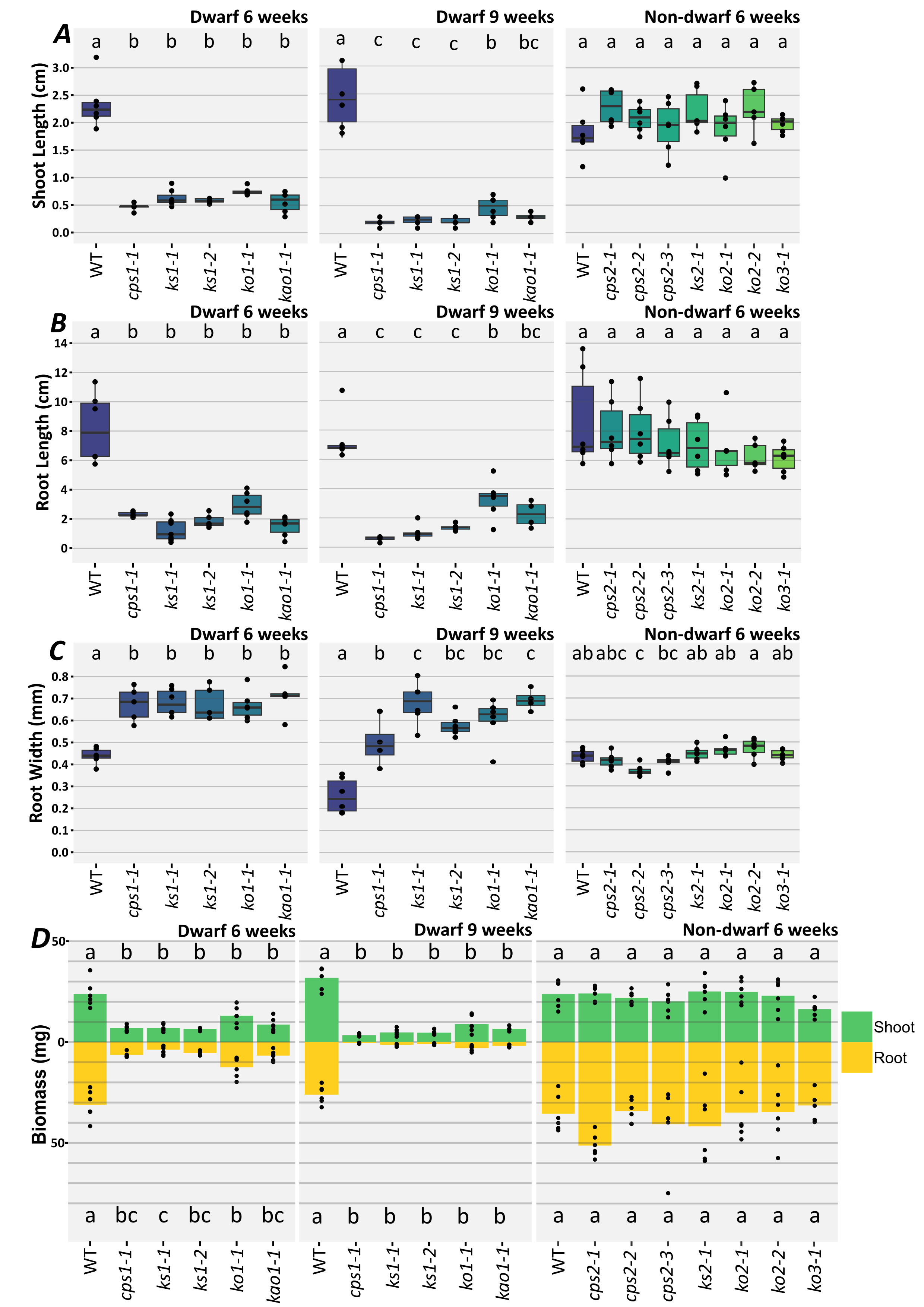

Supplement: Supplementary Figure 6 — Characterisation of shoot and root growth phenotypes of L. japonicus mutants examined in this study. (A) Shoot length of dwarf (left panel and central panel) and non-dwarf (right panel) mutant lines. Significance groups were calculated for each panel (Tukey HSD, p < 0.05) (n ≥ 5 biological replicates. Each replicate represents a single plant). (B) Root length of dwarf (left panel and central panel) and non-dwarf (right panel) mutant lines. Significance groups were calculated for each panel (Tukey HSD, p < 0.05) (n ≥ 5 biological replicates. Each replicate represents a single plant). (C) Root width of dwarf (left panel and central panel) and non-dwarf (right panel) mutant lines. For each plant, root width was determined as the average of 10 individual regions, randomly selected along the root length. Significance groups were calculated for each panel (Tukey HSD, p < 0.05) (n ≥ 5 biological replicates. Each replicate represents a single plant). (D) Fresh root and shoot biomass of dwarf (left panel and central panel) and non-dwarf (right panel) mutant lines. Significance groups were calculated for each panel (Tukey HSD, p < 0.05) (n ≥ 5 biological replicates. Each replicate represents a single plant). [file Image6.tiff]

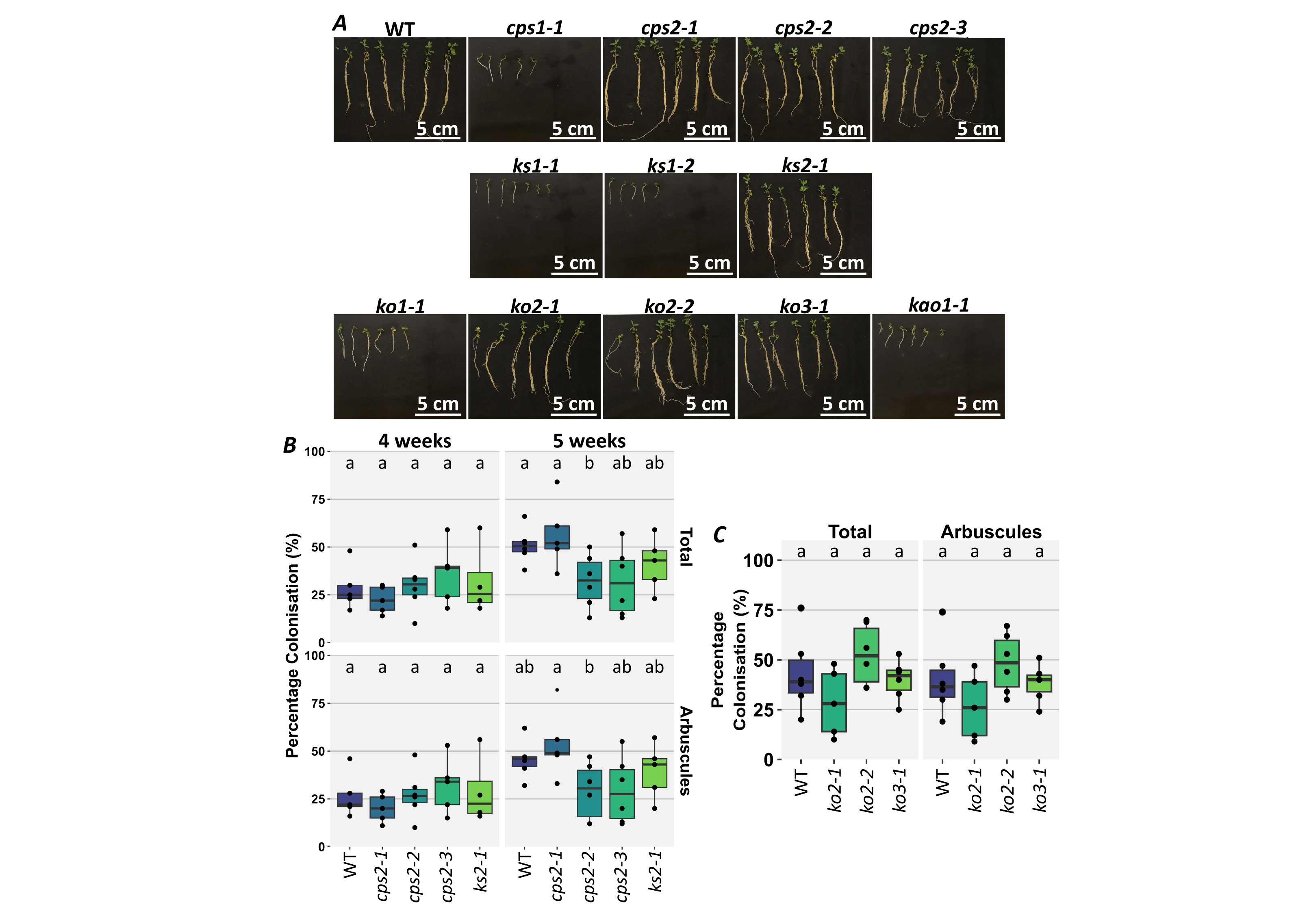

Supplement: Supplementary Figure 7 — Phenotypes of cps2, ks2, ko2 and ko3 mutants (A) Representative images of the growth phenotypes of cps1, ks1, ko1, and kao1 mutants, displaying severe growth phenotypes, compared with mutants in their respective gene homologs. Plants were grown under conditions for arbuscular mycorrhizal colonisation assays and imaged at 6 weeks old. (B) Root-length colonisation of cps2 and ks2 mutants. Plants were assessed at 4 and 5 weeks post-inoculation. Significance groups were calculated for each panel (Wilcoxon Rank Sum Test, p < 0.05) (n ≥ 4 biological replicates. Each replicate represents a single plant). Statistics were calculated independently for percentage total and percentage arbuscule root-length colonisation, and independently for each timepoint. (C) Root-length colonisation of ko2 and ko3 mutants. Plants were assessed at 4 weeks post-inoculation. No statistical differences were observed (Kruskal Wallis Test, p = 0.26 for percentage total root-length colonisation and p = 0.32 for percentage arbuscule root-length colonisation) (n ≥ 5 biological replicates. Each replicate represents a single plant). Statistics were calculated independently for percentage total and percentage arbuscule root-length colonisation. [file Image7.tiff]

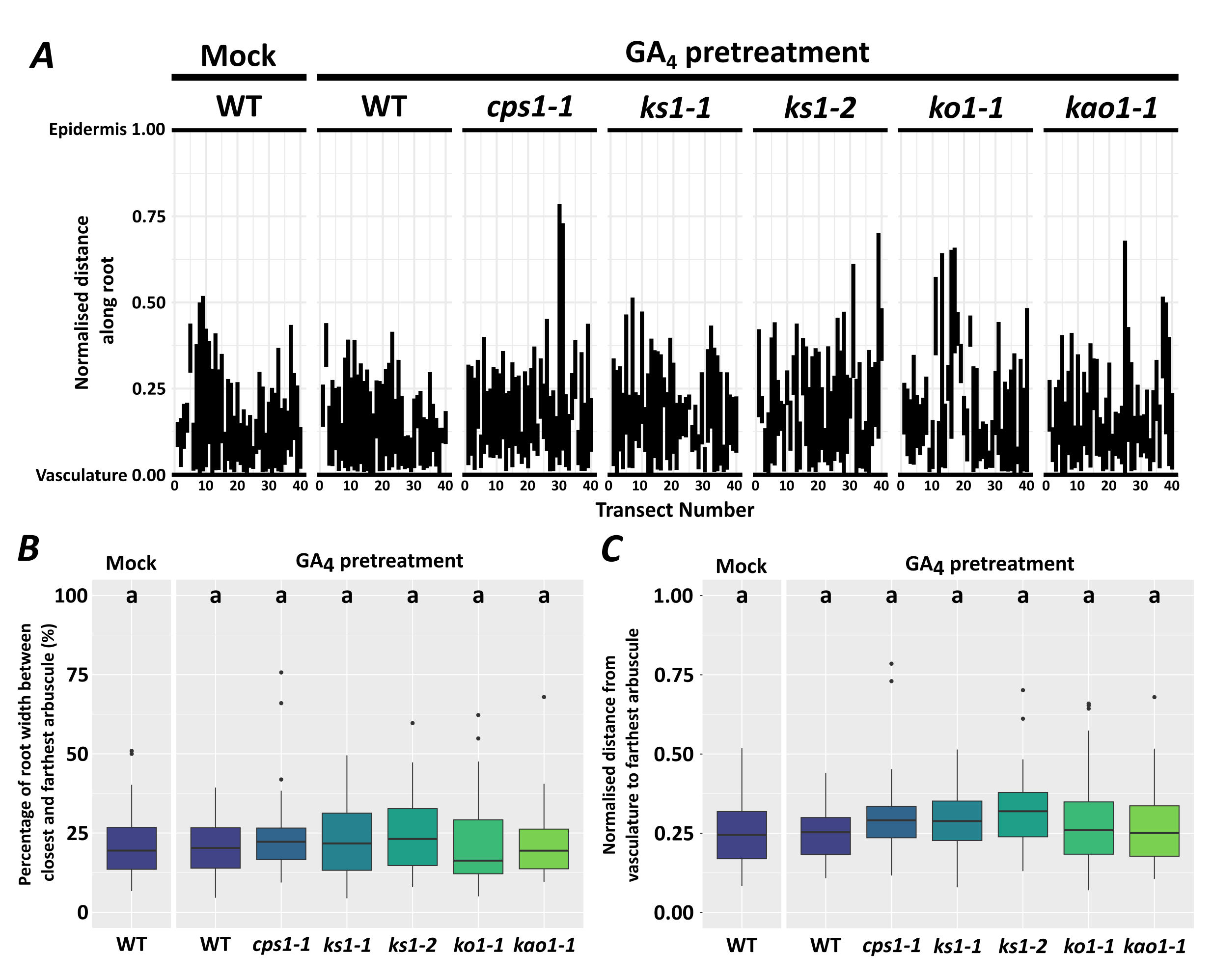

Supplement: Supplementary Figure 8 — Arbuscule distribution is restored in gibberellin-deficient roots pretreated with gibberellin A4. (A) An illustration of the distribution of arbuscules found in WT and mutant lines following pretreatment with gibberellin A4. Each vertical line represents the distance between the closest (close) and furthest (far) arbuscule in each transect. Distances were normalised to the distance between the vasculature and epidermis. (B) The distance between the closest (close) and farthest (far) arbuscule, as a percentage of the root width measured. No statistically significant differences were observed (Kruskal-Wallis test, p = 0.65) (n = 40 transects collected from ≥3 biological replicates). (C) The distance from the vasculature to the farthest arbuscule, normalised to the root width measured. No statistically significant differences were observed (Kruskal-Wallis test, p = 0.11) (n = 40 transects collected from ≥3 biological replicates). [file Image8.tiff]
