# Supplementary material for: Gibberellin biosynthesis in Lotus japonicus regulates arbuscule distribution, but not overall colonisation by arbuscular mycorrhizal fungi
Source: Front Plant Sci. 2026 Mar 20;17:1772317. doi: 10.3389/fpls.2026.1772317 (PMC13047161; doi:10.3389/fpls.2026.1772317)
Supplement: Supplementary file 9 [file Table1.docx]

| **Gene Name** | **Gifu v1.2 Identifier** | **Allele Name** | ***LORE1* Plant ID** | **Source** |
| --- | --- | --- | --- | --- |
| *CPS1* | *LotjaGi1g1v0718300* | *Ljcps1-1* | L9070 | National Agriculture and Food Research Organization |
| *CPS2* | *LotjaGi1g1v0228700* | *Ljcps2-1* | 30141373 | Lotus Bank |
| *CPS2* | *LotjaGi1g1v0228700* | *Ljcps2-2* | 30124554 | Lotus Bank |
| *CPS2* | *LotjaGi1g1v0228700* | *Ljcps2-3* | 30131534 | Lotus Bank |
| *KS1* | *LotjaGi3g1v0132900* | *Ljks1-1* | 30034878 | Lotus Bank |
| *KS1* | *LotjaGi3g1v0132900* | *Ljks1-2* | 30098386 | Lotus Bank |
| *KS2* | *LotjaGi3g1v0133000* | *Ljks2-1* | 30115281 | Lotus Bank |
| *KO1* | *LotjaGi3g1v0002700* | *Ljko1-1* | 30030447 | Lotus Bank |
| *KO2* | *LotjaGi3g1v0002600* | *Ljko2-1* | 30116249 | Lotus Bank |
| *KO2* | *LotjaGi3g1v0002600* | *Ljko2-2* | 30119097 | Lotus Bank |
| *KO3* | *LotjaGi3g1v0002800* | *Ljko3-1* | 30068387 | Lotus Bank |
| *KAO1* | *LotjaGi6g1v0265700* | *Ljkao1-1* | 30012852 | Lotus Bank |

**Supplementary Table 1. IDs of *LORE1* lines used in this study**
