# Supplementary material for: Gibberellin biosynthesis in Lotus japonicus regulates arbuscule distribution, but not overall colonisation by arbuscular mycorrhizal fungi
Source: Front Plant Sci. 2026 Mar 20;17:1772317. doi: 10.3389/fpls.2026.1772317 (PMC13047161; doi:10.3389/fpls.2026.1772317)
Supplement: Supplementary file 10 [file Table2.docx]

**Supplementary Table 2. Primers used for genotyping.**

| **Site** | **Forward Primer Sequence** | **Reverse Primer Sequence** |
| --- | --- | --- |
| *CPS1-1* | ACGCAGGGGCAACCACAATTTGAA | TGGAGGATGACACACATGAGGAGCA |
| *CPS2-1* | CGCAAGCGGTGACAGGAATGTTCA | GCACGTCCAATGCGTAAGCCACCT |
| *CPS2-2* | GTTGGGTAAGAGGGGTTGGCGGTG | TGGTTTTGGCCCATGCAAGTCTCA |
| *CPS2-3* | AGCCTGCCAAAGAGTCTGGTCTACACTT | CTTGCATGGGCCAAAACCACATCA |
| *KS1-1* | CCCCACCACCTGCGCAATGGCA | CGCGCCGGAGAAGTAACAATAGGCCA |
| *KS1-2* | CATTGCGCAGGTGGTGGGGTCTAA | GCAGCTGTTTTTCAGCACCTTAACAATGC |
| *KS2-1* | CAACAGCAGCTGCATTTCAGCACCTTA | AAATGCCATTGCACAGGTGGTGGG |
| *KO1-1* | CCGTGACACGAATCTCTTGGCAGA | TGAGAACGATGAGGGTGAAAGCACCA |
| *KO2-1* | ACCTCCTTGGCAAGCTGGGCAG | CCGCTAGATCACCCGTCCACGCCA |
| *KO2-2* | CAACAATCTTTGTGAAATGGAGGGAGTACA | TCGGAGCCAATGCACAGGTTGGT |
| *KO3-1* | AAGAGCAACTAACCTCTTTGGC | CAGTACTGAGTTGTGTTGCTGT |
| *KAO1-1* | GCTCATGCAATCCTCCGCGATTGT | GGGCCATGATTCCGCTTCCTATGC |
