# Supplementary material for: Gibberellin biosynthesis in Lotus japonicus regulates arbuscule distribution, but not overall colonisation by arbuscular mycorrhizal fungi
Source: Front Plant Sci. 2026 Mar 20;17:1772317. doi: 10.3389/fpls.2026.1772317 (PMC13047161; doi:10.3389/fpls.2026.1772317)
Supplement: Supplementary file 11 [file Table3.docx]

**Supplementary Table 3. Recipe for the production of 10x B&D media.** *Filter sterilised. pH adjusted to pH 5.8–6.2.

| **Chemical species** | **Mass (g)/l** | **Stock concentration (x)** | **mL stock to make 1L 10x B&D Medium** |
| --- | --- | --- | --- |
| **A** | | | |
| CaCl2.2H2O | 147.02 | 1000 | 10 |
| **B** | | | |
| KH2PO4 | 27.22 | 1000 | 10 |
| KCl | 22.37 | 1000 |  |
| **C** | | | |
| Fe(III)-citrate* | 2.45 | 1000 | 10 |
| **D** | | | |
| MgSO4.7H2O | 61.62 | 1000 | 10 |
| K2SO4 | 87.13 | 1000 |  |
| MnSO4.H2O | 0.17 | 1000 |  |
| H3BO3 | 0.12 | 1000 |  |
| ZnSO4.7H2O | 0.14 | 1000 |  |
| Na2MoO4.2H2O | 0.024 | 1000 |  |
| CoCl2.6H2O | 0.024 | 1000 |  |
| CuSO4.5H2O | 0.050 | 1000 |  |
| **E** | | | |
| KNO3 | 151.65 | 1000 | 10 |
| NH4NO3 | 200.10 | 1000 |  |
