# Supplementary material for: Gibberellin biosynthesis in Lotus japonicus regulates arbuscule distribution, but not overall colonisation by arbuscular mycorrhizal fungi
Source: Front Plant Sci. 2026 Mar 20;17:1772317. doi: 10.3389/fpls.2026.1772317 (PMC13047161; doi:10.3389/fpls.2026.1772317)
Supplement: Supplementary file 12 [file Table4.docx]

**Supplementary Table 4. Names and sources of genomes used in this study for phylogenetic analyses.**

| **Genome** | **Reference(s)** | **Source** |
| --- | --- | --- |
| *Arabidopsis thaliana* Araport11 | (Cheng et al., 2017) | https://phytozome.jgi.doe.gov/pz/portal.html |
| *Hordeum vulgare* r1 | (Beier et al., 2017; Mascher et al., 2017) | https://phytozome.jgi.doe.gov/pz/portal.html |
| *Lotus japonicus* Gifu v1.2 | (Mun et al., 2016; Kamal et al., 2020) | https://lotus.au.dk/ |
| *Lotus japonicus MG20_v3.0* | (Sato et al., 2008; Mun et al., 2016) | https://lotus.au.dk/pw |
| *Lupinus albus* | (Hufnagel et al., 2020) | https://www.whitelupin.fr/index.html |
| *Marchantia paleacea* | (Radhakrishnan et al., 2020) | https://www.polebio.lrsv.ups-tlse.fr/symdb/web/  Access provided by Cyril Libourel |
| *Marchantia polymorpha* v3.1 | (Bowman et al., 2017) | https://phytozome.jgi.doe.gov/pz/portal.html |
| *Medicago truncatula* Mt4.0v1 | (Tang et al., 2014) | https://phytozome.jgi.doe.gov/pz/portal.html |
| *Oryza sativa* v7.0_JGI | (Ouyang et al., 2007) | https://phytozome.jgi.doe.gov/pz/portal.html |
| *Physcomitrella patens* v3.3 | (Lang et al., 2018) | https://phytozome.jgi.doe.gov/pz/portal.html |
| *Pisum sativum* | (Kreplak et al., 2019) | https://urgi.versailles.inra.fr/Species/Pisum |
| *Solanum lycopersicum ITAG4.0* | (Hosmani et al., 2019) | https://phytozome-next.jgi.doe.gov/ |
| *Sorghum bicolor v3.1.1* | (McCormick et al., 2018) | https://phytozome.jgi.doe.gov/pz/portal.html |
| *Zea mays PH207 v1.1 (Maize PH207)* | (Hirsch et al., 2016) | https://phytozome.jgi.doe.gov/pz/portal.html |
